# Been There, Done That, and Now I’m Giving Back: Perspectives from Mentors and Administrators in Community Violence Intervention Programs

**DOI:** 10.1007/s11121-026-01877-4

**Published:** 2026-01-16

**Authors:** Kristian Jones, Jarvis Duckworth, Chloe Fetters, Biruktawit Galoro, Ali Rowhani-Rahbar

**Affiliations:** 1 Center for Firearm Injury Prevention, University of Washington, Seattle, WA USA; 2 School of Social Work, University of Washington, Seattle, WA USA

**Keywords:** Mentoring, Firearm violence prevention, CVI programs, Qualitative framework

## Abstract

Black young people are at high risk for firearm homicide in the USA. A promising intervention for mitigating firearm-related injuries and deaths is mentoring provided by individuals with specific lived experiences with firearm violence and trauma (e.g., credible messengers, violence interrupters) in community violence intervention (CVI) programs. The purpose of this study was to identify the factors through which mentoring interventions in CVI programs could cultivate protective factors (e.g., social support) that prevent interpersonal firearm violence. Semi-structured interviews were conducted with 20 mentors and program administrators in CVI programs in Washington State to examine their insights on the components of mentoring interventions that could work towards preventing firearm violence. A framework was developed through the use of a constructivist grounded theory methodology to capture the components within mentoring interventions in CVI programs aiming to prevent firearm violence among marginalized young people in communities disproportionately impacted by community firearm violence. Implications for both mentoring and CVI research are outlined in the manuscript.

The rising prevalence of firearm violence in the USA has disproportionately affected Black young people, a crisis rooted in systemic policies and societal disparities (Buggs et al., 2023). Between 2014 and 2020, the number of firearm-related deaths among Black young people worsened, escalating this cycle of violence (Wolf et al., 2024). A study conducted by Mariño-Ramírez et al. (2022) found Black youth and adolescents experienced firearm-related deaths at significantly higher rates than any other racial demographic. Specifically, Black young people ages 1–19 accounted for 47.1% of firearm-related fatalities among young people ages 1–19 despite only accounting for 15% of young people in this age group. Such levels of violence victimization among Black young people are caused by multifaceted factors, thus calling for a multidimensional approach Buggs (2022). One intervention that holds promise is mentoring, specifically mentoring provided by trusted individuals with lived experiences with firearm violence, providing guidance to young people in communities most impacted by firearm violence (Wical et al., 2020).

## Literature Review

### Providing Mentorship to Reduce Firearm Violence

Provisions of mentorship in firearm violence prevention and intervention programs extend across various institutions, including community organizations, hospitals, and schools (Jones, et al., 2021). Mentoring interventions in community violence intervention (CVI) programs aim to provide a system of support for young individuals who may experience high risks of firearm violence, creating opportunities for connection and recovery. Through utilizing mentoring interventions within CVI programs, community organizations are able to provide young people with the support needed to address basic needs, challenge ways of thinking, handle conflict, and change social norms with the ultimate goal of preventing firearm violence altogether.

### Effectiveness of Mentorship in CVI

The research examining the effectiveness of CVI programs that utilize mentoring to reduce firearm-related outcomes among vulnerable young people has been mixed. For example, Cheng and colleagues (2008) illustrated no significant differences in risk for injury between young people who participate in CVI programs that include mentoring interventions and young people who do not utilize services at all. In this study, mentoring was integrated into a case management contact model provided to assaulted young people via in-person services or by telephone over 4 months. A 6-month follow-up assessed how the risk behaviors, attitudes about violence, mental health, and injury history of the young people receiving the services.

Some CVI programs such as Advance Peace (AP) and Cure Violence (CV) have proven effective in reducing firearm-related outcomes in certain settings through utilizing specific interventions that use mentoring and broader social support (Butts et al., 2015; Corburn et al., 2021). The mentoring in the AP intervention consists of one-on-one interactions with young people most at risk of firearm violence, meeting with credible messengers 7 days a week. These interactions include in-person visits, phone calls, and texting for at least 18 months. The study reported that the young people who participated in the program had reduced firearm assaults along with improved social connections and life skills. Meanwhile, CV’s mentoring intervention utilizes Violence Interrupters who focus on identifying and interrupting violence by building rapport with high-risk young people and responding to critical incidents when they occur, while Outreach Workers meet with these young people several times a week to connect them to various types of resources. The CV model emphasizes ongoing relationship building rather than a strict timeline when working with young people. The CV model is utilized in programs across several cities in the USA, and the results of evaluations in different sites have found decreases in homicides and non-fatal shootings in some sites but not others. Overall, while the evidence is mixed on the effectiveness of mentoring interventions in CVI programs striving to prevent firearm-related outcomes, there are promising findings that underscore the importance of these types of interventions for young people.

### The Current Study

While some research suggests that CVI programs can be impactful in reducing firearm violence and other firearm-related outcomes, less is known about what components of the mentoring interventions within these community programs are employed for preventing community firearm violence, especially among Black young people. The purpose of this study was to examine the components of mentorship in CVI programs used to prevent firearm violence among vulnerable young people. Using semi-structured interviews with current mentors and administrators in CVI programs in Washington state, this study identified the components of mentoring interventions within CVI programs that are used to prevent firearm violence.

## Methods

### Participants and Procedure

Semi-structured interviews were conducted with 20 mentors and administrators in CVI programs to understand their experiences working in these programs and, importantly, to develop a framework based on the key components they identified, aimed at guiding ongoing efforts to prevent interpersonal firearm violence. The participants were current mentors and administrators in several CVI programs across the state of Washington. The first author relied on strong relationships that they established with CVI programs to recruit mentors and administrators who would be interested in discussing their experiences mentoring, with the ultimate goal to prevent firearm violence. Of the 20 participants, 14 of them were administrators, and 12 of those administrators had experience directly providing mentorship to young people in CVI programs. Considering the disproportionate impact of firearm violence felt by Black young people, emphasis was placed on mentors who have worked with Black communities in some capacity. Because of the location of the lead researcher and research team and the emphasis of the grant supporting this research, focus was placed upon recruiting participants in Washington state.

To participate in the study, participants had to fulfill the following criteria: (a) be a mentor, staff member, or administrator currently working within a firearm prevention program in Washington state and (b) expressing willingness to consent to participate in the study, as well as completion of at least one in-depth, semi-structured individual interview regarding their experiences and insights on providing mentorship to a young person in a firearm violence prevention program. Prospective participants were excluded if any of the following criteria applied: (a) failure to meet all inclusion criteria or (b) involuntary suspension or expulsion from a firearm violence prevention program.

### Data Collection and Analysis

Given that mentors and administrators were located across Washington state and the demanding nature of their jobs providing direct services to marginalized communities, all interviews took place exclusively via video calling (e.g., Zoom) between July 2024 and March 2025. Interviews lasted between 45 and 90 min, and all participants were compensated $50 for their participation and were allowed to withdraw from the interview at any time without penalty. Interviews commenced only once IRB approval was granted by the authors’ institution.

The transcriptions of the audio recordings of all 20 interviews were verified by the first and second authors listening to each recording, making any necessary corrections, and anonymizing each interview in the Otter transcription software. Memoing and coding occurred throughout the data collection and data analysis process and began after the first interview was conducted to align with the principles of constructivist grounded theory (CGT; Charmaz, 2014). Memoing and critical reflection throughout the data collection process were essential as CGT acknowledges the role of the researcher’s positionality and lived experiences in the process of the co-creation of knowledge in the study (e.g., being a Black researcher with previous experience as a mentor and having lived experience of being impacted by firearm violence).

Coding was conducted by the first author and consisted of two phases: (a) initial and (b) focused coding, while utilizing the constant comparative method to ensure that reflection of positionality was identified in the coding process. Initial coding consisted of analyzing mentors’ and administrators’ backgrounds and motivations to prevent firearm violence in CVI programs through mentorship, identifying the key characteristics and experiences that promote positive outcomes in mentorship relationships, examining how race and culture impact the mentoring relationship, and uncovering how they work with the young person’s family and social network within the intervention. This stage of the coding process utilized “in vivo” coding to emphasize the participants’ words when coding the data. The next stage of coding, focused coding, consisted of the identification of major sub-themes that stemmed from the refinement of initial codes and building more expansive categories. Data were categorized to be the most representative of all of the mentors’ and administrators’ experiences as it related to their motivations and lived experiences related to firearm violence and trauma before the mentoring relationship starts, essential components of the CVI mentoring intervention (e.g., addressing the basic needs of the young person). While discussing their experiences, mentors acknowledged the broader macro factors that contribute to interpersonal firearm violence in their community. Through focused coding, data were synthesized into larger themes that captured the process occurring in the CVI mentoring intervention from the perspective of the mentors and administrators (Charmaz, 2014). Theoretical saturation was reached after 17 interviews, and three more interviews were conducted to check for outlier themes. Although inter-coder reliability was not feasible for this study, the first author reviewed the codes with the second author and conducted member checking interviews with five participants who expressed interest in having a follow-up conversation to review the findings and preliminary framework. All the participants expressed approval of the findings, specifically stating that the preliminary framework captured the work that they did on a daily basis to promote safety in their community. These participants were from four different CVI programs from different areas of the state.

### Positionality Statement

Considering this study utilized CGT, the first author’s intersecting identities and positionality had a profound impact on the data collection and data analysis of the study. The first author, a Black cis-gendered male, lost a friend and fraternity brother to firearm violence in 2018, has extensive experience working with youth as a mentor and mental health clinician, and is a faculty member who studies how youth mentoring can promote positive outcomes among Black youth. The second author is a cisgender Black male who works as a student and research assistant with lived experience in communities affected by violence, drawing on their background in CVI work. His approach to this project is shaped by the insights and mentorship he gained through active engagement in this field. Growing up in underserved neighborhoods, he witnessed firsthand the gaps in guidance and support available for young Black individuals. This project is rooted in his commitment to amplifying the importance of mentorship, healing, and community safety, ensuring that young Black people can access a promising future.

**Fig. 1:**
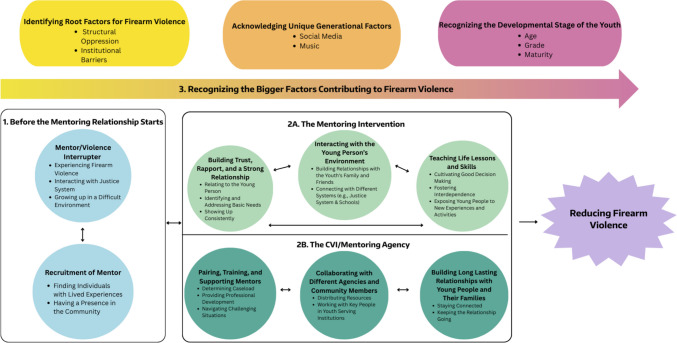
Mentoring interventions in CVI program framework

The third author is a white, cisgender woman who is an undergraduate student studying Social Welfare and grew up in Orange County, CA. The fourth author is a Black American and East African woman obtaining her BASW and has personally experienced firearm violence, having lost a neighbor and several community members. She has worked with organizations that advocate for and work to prevent firearm violence among young people. The fifth author is a Middle-Eastern cis-gendered male who has been conducting research on firearm violence for 13 years and served as a mentor for the first author for the past 2 years in his capacity as a faculty member and the director of a research program in this area. Memo writing was vital throughout data collection and data analysis and helped the authors, specifically the first author, keep track of their initial reactions and reflections throughout the research project. These memos were then integrated into the final framework created from the data (see Fig. 1; Charmaz, 2014).

## Results

Study participants’ perspectives were used to identify the elements they considered most important for reducing firearm violence among youth (see Fig. 1). The findings outlined below highlight that this process begins even before the mentoring relationship is established. Participants then reflected on the mentoring intervention itself, as well as the agency which delivers the intervention. All of these aspects should be understood within the broader context of the systemic factors of firearm violence.

### Before the Mentoring Relationship Starts

#### Individuals Reflecting on Motivations to Prevent Firearm Violence

A large majority of the mentors and administrators described personal or vicarious experiences (e.g., family, friends) involving exposure to firearm violence in their community, gang affiliation, overcoming addiction, and/or interaction with the justice system. In fact, all of the participants in this study had the misfortune of being exposed to firearm violence, whether it was gun violence directed at them individually or living in a community where firearm violence was a common occurrence. These were pivotal experiences that not only shaped their motivation to become a mentor or an administrator in a CVI program but also shaped how they went about providing mentorship to young people in their community. One mentor (#19) explained how being from the community and losing close friends to firearm violence motivated them to serve their community as a mentor,

I have multiple homies that have died…everybody’s willing to die for homies, but you want to live for them. So, I guess it’s that aspect of it, being able to see their kids. Because a lot of my homies’ kids are on my caseload, which is crazy.

#### Recruiting Mentors

CVI programs that are using mentoring interventions to prevent firearm violence actively seek out and recruit these individuals with these specific lived experiences. One administrator (#11) described how essential it was to find mentors who could relate on a deeper level to what the young people in the program were experiencing:

…in order to really understand the life of someone who’s been incarcerated and what they’re going through, or even to know the of gun violence…they really have to have the knowledge of that. Not read it in some book or I have a friend of a friend of a friend… because it’s just different.

##### Using Connections in the Community

CVI programs in this study reported utilizing their connections in the community to recruit individuals who would be a good fit for this type of mentorship. When discussing the importance of being in community to find mentors, an administrator (#14) detailed, “That’s, where you’re going to find them, because there’s no website where you can go to find people with lived experience…that doesn’t exist. So, you really gotta talk to people in the community.” Another administrator (#4) highlighted that many of the mentors in their organization were once youth receiving services in the CVI agency. “…I’ll say half of our staff came from our program…that’s one a win for us, but it’s also a win for the work, because they’re from the neighborhood.” While most recognized that the mentors’ lived experience and connection to firearm violence was essential to effective mentoring to prevent firearm violence, many discussed the difficulty that comes with recruiting mentors from a specific demographic. One administrator (#6) explained:

Yeah, it’s really difficult because it’s such a unique set of criteria…we’re looking for people who grew up in the life, who’ve been incarcerated, who’ve had substance abuse issues, overcame all that, and now want to turn it around and use their experiences to help other people. It’s not like they’re lining up on the outside the door, waiting to get in.

### The Mentoring Intervention/Relationship

#### Relating to the Young Person

As mentioned earlier, in some capacity, the mentors and administrators doing this work have lived experiences with firearm violence in their community. This could include being a former gang member or spending time in the legal system. This could also include living in a community impacted by high rates of gun violence and incarceration and still graduating high school, yet never being incarcerated or gang-affiliated. Participants discussed how their lived experiences directly allowed them to identify with what the young person was going through and connect with them on a deeper level. One mentor (#5) described a time one of his mentees expressed their appreciation for how he could relate to their experience:

… I was talking to a young brother at the detention center, and he said to me, “we really appreciate you coming here.”, And I said, why? He said, “Because of everyone that comes here and talks to us, you’re the only one that understands what it’s like.

##### Acknowledging Race, Ethnicity, Gender, and Culture

Participants discussed the importance of acknowledging the identity of the young, specifically their cultural identity, and discussed ways in which those identities shaped conversations in the relationship. This was especially important when the mentor shared those same identities themselves (e.g., being a Black male from the same city). When discussing the importance of having mentors reflect the identities of the community, one administrator (#15) explained, “I’ve definitely seen them be able to relate with clients, being discriminated against themselves, and share that experience, but also share it in a way where they’re encouraging healthier ways to address it when it happens.”

##### Having a Presence in the Community

Many of the participants in the study discussed the importance of having a presence in the community they served, and how their reputation allowed them to connect with the youth and relate to them on a deeper level. One mentor/administrator (#9) explained:

A lot of trust comes from me knowing people. A lot of times when I meet a young person, I’m gonna ask him, “Where you from, Where’d you grow up? Who do you be around?” I’ll usually be like, “Hey, you know, such and such? That’s my boy. Ask him about me.

##### Listening and Being Non-Judgmental

Listening in a non-judgmental manner was highlighted universally among the participants as an essential component for connecting with their mentee on a deep level during the relationship. Particularly, participants emphasized that young people often feel judged in other areas of their life, and creating a space for the young person to feel heard without judgement was critical. One mentor (#3) explained their approach to building deep bonds with young people who have experienced the most trauma, “Just being open to listening and listening without judgment, listening without waiting to respond, thinking about how I’m going to respond to this, because I’m a mentor, and I gotta have some wisdom…”. In the relationship-building stage of the relationship, mentors leaned on their colleagues and the broader CVI program to provide training and collaboration on how to best serve the young person. Participants described this type of support as receiving professional development training (e.g., one program provided training on motivational interviewing), and in some cases reassigning the mentor to a young person with whom they have a better fit than their colleagues.

#### Showing Up Consistently

In addition to being relatable, participants underscored the importance of consistency and stressed that constantly showing up allowed the young person to lower their guard, which ultimately led to trust within the relationship. Mentors in particular discussed the importance of staying true to your word and consistently showing up despite the initial apprehension from the young person. One mentor (#18) reflected on a time they built a deep connection with a young person despite a rocky start to the relationship:

I met this kid; she fired me day one. Threw a pillow at me, fired me, told me she never wants to see my face again. And I was like, “Okay, I’ll see you next week.” And I came back, and then I kept trying to talk to her, and she was like, “I don’t give a f***”. And I was like, “That’s cool, bro”, but I just kept showing up….And by the end of it, she gave me a little Dobby figurine from Harry Potter, because she knew Dobby was my favorite.

Participants also highlighted that the amount of time it took to build trust often varied by the needs of the young person and how often the mentor and agency were in contact with the person. Mentors highlighted that they could contact a young person up to three times a week when a young person first enters the program to build that rapport and ensure their needs are being met, especially if the young person is gang-affiliated or has high involvement with multiple systems. Moreover, many expressed that a connection could be built instantly with some young people due to having shared lived experiences around firearm violence and trauma, but with others, the process could take between 6 months and a year for the mentors and the agency to prove they were worthy of being trusted.

#### Addressing Basic and Essential Needs

Participants in this study emphasized that mentoring to prevent firearm violence is impossible without addressing basic and essential needs (e.g., housing, food, safety) of the young person. This can include a variety of different resources including but not limited to: providing a young person with clothes, helping a young person go through the process of getting their driver’s license, and helping a young person apply for a housing program. One of the more common ways mentors addressed the basic needs of the young people in their program was by providing transportation for the youth so they can navigate their community safely. One administrator/mentor (#4) described how their organization provided transportation to young people in their program to help them avoid potentially dangerous situations. Another mentor (#8) described the importance of transporting their mentee to important meetings that would be hard for them to attend otherwise, stating:

If you have a client that has probation appointments, I always try to offer to take them there, to make sure that they get there. Lots of times, parents are working or parents aren’t involved, so…mentors help do that and get them there.

### Interacting with the Young Person’s Environment

In addition to providing tangible resources to the young people in their respective programs, participants in this study stressed the importance of coordinating with important people in the youth’s environment, whether that be their immediate family and friends or people in the other systems that have a large impact on the young person’s everyday life. In this aspect of the mentoring intervention, the mentor and the CVI agency work in tandem to support the young person and ensure they have the support they need to address any environmental hurdles that may make them particularly vulnerable to firearm violence.

#### Building Relationships with the Youth’s Family and Social Network

Participants detailed how critical it was to coordinate with the young person’s family and broader social network in the mentoring intervention. Mentors and CVI programs often support families by addressing their basic needs and, in dire situations, help relocate families when their safety is in jeopardy. Many participants underscored that, while they work primarily with the young person in the program, the family is a vital aspect of the service provision as well.

##### Using Family Members as Assets in the Relationship

Participants discussed the importance of leveraging the young person’s family as an asset in the relationship. One mentor/administrator (#4) described a complex situation of coordinating with a sibling of one of the young people in the program,

…we actually have a kid who whose brother is gang related, but he doesn’t want his younger brother to be involved, and so our my one of my staff has a good relationship with the brother, and he’s working with the brother to say, “Hey, man, I don’t want him on the streets either. So, if you can just work with me to make sure he doesn’t get involved in that…”.

#### Advocating for and with the Youth

In addition to working with the youth’s family and peers, mentors and CVI programs also constantly interact with people within systems the young person is involved with while they are in the program, while connecting them to people and resources that can be used to navigate these systems (e.g., a psychologist, lawyer, housing specialist). When working with various systems that impact young people, mentors and administrators explained that they are often advocating for and with the young person to make sure their needs are met. One example of advocacy that can take place between a mentor and the young person is making sure the young person understands paperwork they are required to sign when they are involved with the justice system. One mentor (#9) discussed a time they made sure their mentee understood the paperwork by translating it in a way they understood it.

…it’s really about… transforming it into their language for real, even with their attorneys, you know, I sit down with their attorneys and the client a lot of times to go over paperwork, to go over plea deals, so they know what they’re signing.

##### Interacting with the Justice System

Many of the participants explained that most of their youth interact with the justice system in some capacity and detailed how this led to their own involvement with the system to support their mentee. One mentor (#8) explained how their organization includes important people in the juvenile justice system in their meetings with their mentee, “We have a caseload meeting where probation officers and other people are involved, and we go over our cases with them, you know, just to bounce off ideas and see how we can, you know, better help this youth.” Another mentor (#9) explained how having a good relationship with the mentee’s probation officer and legal team allowed them to advocate for the young person and support them when they were at risk of being reprimanded for a violation:

We know probation. They’ll reach out to us like, “Your boy went out of range and went too far out his house, tell him he can’t be doing that”…instead of just violating them right away, a lot of times they will let me have that conversation [with the young person]

##### Coordinating with the School System

Another system participants consistently identified as being essential to interact with was the school system. One administrator (#20) detailed having to advocate for their mentee in school and provide context for why the young person was missing class:

It’s very critical that that we collaborate with school systems, because they have our kids majority of the time, and so understanding and training them, giving them awareness…like… [this youth] is the oldest in the family, and their taking care of their little siblings. That’s why their truant first and second period …

Many of the CVI programs in this study used their connections to different organizations and systems to provide the young person access to resources to help navigate the various systems impacting their daily life. The mentor is often working alongside the broader agency to support the young person and their family to make sure they are receiving holistic and robust support.

### Providing Skills and Teaching Lessons

#### Cultivating Decision Making Skills

In addition to building a strong relational bond and interacting with the systems and individuals impacting their mentee, participants detailed the different ways they promoted sound decision-making skills with their mentee. Many were adamant that helping the young person in their program make wise decisions was a critical aspect of the mentoring intervention *successfully* intervening and preventing firearm violence. Some mentors talked about how they cultivated these decision-making skills in one-on-one conversations, while other mentors emphasized how getting the mentee to interact with other young people in the program who were striving to do better was also an impactful way to cultivate decision-making as well.

##### Seeing a Future

Some mentors discussed the importance of helping the young person see a future for themselves as a critical aspect of cultivating their decision-making skills, particularly in regard to making decisions to avoid situations that could lead to firearm violence or other dire consequences. For example, one participant (# 9) explained, “…when you have a vision for something, it makes it easier for you to be like, “Nah, I’m cool. I don’t want to do that”…and I see this, like changing [their] mindset.”

##### Getting Them to See the Magnitude of Their Actions

Participants also discussed the importance of getting young people to be able to grasp the severity of their actions, those both in the past and future. One mentor (#5) detailed how they emphasized the magnitude of a young person murdering another human being:

And the most unfortunate thing about him and his situation is he really didn’t understand the magnitude of what he had done and how he had really impacted not only the life that he took, not just that gentleman’s family, but also the people that worked at that, at that at that place of business, they were traumatized as well.

#### Fostering Interdependence

Both mentors and administrators stressed the importance of fostering skills specific to helping the young person take the lead on advocating for themselves and only utilizing the program when needed. One way participants promoted interdependence was teaching young people how to navigate complex systems (e.g., the justice system) so they would not have to rely on the program forever. Participant 17 explained the importance of teaching young people about navigating complex systems:

…I’ve noticed in this work you’re not really doing as good as the job you should if you’re not really teaching them that life skill to take away when you’re no longer there. So, if they can’t navigate with these systems afterwards, how much did you really do?

### Recognizing the Bigger Factors Contributing to Firearm Violence

While all of the participants believed they were positively impacting their community and reducing firearm violence, all of them acknowledged that their programs faced macro factors (e.g., white supremacy, racism, generational trauma) beyond their control.

#### Identifying Systemic Root Factors for Firearm Violence

The majority of the participants discussed larger systemic issues that were either the root causes or larger contributors to firearm violence in their community. These contributors ranged from macro factors to racism, sexism, poverty, and white supremacy. Participant 12 referenced toxic masculinity as a critical root cause of firearm violence that must be acknowledged:

…I do root cause analysis on the gun violence aspect, young men from the time… they’re taught from the youngest age not to cry, not to share no emotions…So, when you think their first response is aggression…they’re being taught that from a young age, and we’re not putting this connection together…

##### Identifying Lack of Structural Support

The majority of participants expressed their frustration with the lack of resources in communities plagued by firearm violence, and discussed at length the importance of providing people with resources so they did not have to resort to violence for survival. One administrator (#15) explained:

The biggest thing I see with gun violence is… the resources are really slim to none in the in the community, kids are not carrying guns and stealing or selling drugs or doing the activities where we feel like we need to have a gun to be safe, because they have a full refrigerator full of food and they have a safe place to live, it’s usually the opposite.

#### Acknowledging Unique Generational Drivers of Firearm Violence

##### Identifying the Impact of Social Media

Several mentors highlighted the importance of considering how specific generational factors that their mentees had to contend with are much different than they were when they were younger. Specifically, participants discussed how prevalent social media is among young people now and how it can lead to young people making bad decisions based upon trying to maintain or create an online presence. One mentor (#18) reflected, “And I don’t know where that shift happened, and so I don’t know if it has to do with how prevalent social media is, because it wasn’t as prevalent when I was growing up, versus how it is now.”

##### Considering the Role of Music 

A few mentors identified music, specifically hip-hop/rap music, as a unique contributing factor to firearm violence that was specifically different with young people now than in other generations. One administrator (#10) declared, “The cycle just keeps going, and it’s growing…I contribute that to music somewhat, because the music does so much, it’s so influential, and it goes across the globe, and has people doing things, that the music is saying do.”

#### Acknowledging the Age of the Youth Within Their Lifespan Development

Mentors and administrators discussed the importance of considering the youth’s age and their developmental considerations within their lifespan development. This informed how mentors discussed certain topics related to firearm violence and general programming in their respective organizations as the young person’s age, grade level, and maturity all had to be taken into consideration. One mentor/administrator (#4) explained why he thought middle school age youth were the perfect demographic to focus on for prevention, “ So strategically, I think middle school is a prime time for mentorship…this is the prime age where the body’s rebooting itself, the brain is relearning itself…”.

## Discussion

Findings from this study stress the importance of identifying mentors with shared experiences as young people, whether that be coming from the same community or having shared identities, so that mentors are equipped to relate to experiences related to firearm violence. Further, participants identified the importance of mentors being relatable, addressing the basic needs of the young person, connecting with their social network, teaching life lessons, and recognizing macro factors in firearm violence in mentoring interventions in CVI programs. The findings underscore how important it is for CVI programs to have a presence in their community to find talented individuals who can provide this specialized type of mentoring (Hureau & Papachristos, 2024).

The importance of mentors being able to relate to their mentees has been captured in youth mentoring literature (Lester et al., 2019; Spencer et al., 2020). Specifically, Lester and colleagues discussed the importance of experiential empathy (i.e., when mentors can provide relevant lived experiences to connect with the young person) and how experiential empathy is critical to building rapport with young people and connecting with them in an authentic manner. This may be especially essential when providing mentorship on a topic as sensitive and urgent as firearm violence and underscores how essential it is to find mentors with this specific lived expertise and intersecting identities that can relate to young people disproportionately impacted by community firearm violence (Buggs, 2022). Further, the importance of mentor relational characteristics, especially when working with marginalized young people, such as being authentic, showing up consistently, and being non-judgmental, has been underscored within the mentoring literature as well (see Goldner & Ben-Eliyahu, 2021). The importance of organizations recruiting individuals with specific lived experiences into the workforce has been captured in the recent work illustrating the importance of people with lived experiences serving in health service services as well (Jackson-Spierker & Barrows, 2024).

One essential component of the mentoring interventions in CVI programs identified by participants was providing resources (e.g., housing, food, funding, transportation) to young people and their families in order to ensure their basic needs are met. The research is clear that lack of access to socioeconomic resources is associated with higher levels of firearm violence in communities (Barrett et al., 2022; Sanchez et al., 2020). Mentors and administrators in CVI programs addressing the basic needs of both the young people and their families in their programs is an admirable attempt to address some of the root causes of firearm violence. This finding illustrates the importance of CVI programs combining resource allocation with their direct mentoring services to provide holistic support to the young person and their family (Center on the Developing Child at Harvard University, 2021; Masten et al., 2021).

In addition to providing tangible resources to the young people in their respective programs, participants also highlighted the importance of both the mentor and broader CVI program developing a working relationship with other individuals and systems outside of their immediate family impacting the young person in the mentoring intervention, specifically the juvenile justice and school systems. Research illustrates that young people involved with the justice system and/or having issues within their school (e.g., lack of school connectedness) are especially vulnerable to be exposed to firearm violence (Pulavarthi et al., 2024; Swanson et al., 2022; Zheng et al., 2023). The importance of mentors collaborating with other important people and systems in the young person’s life to provide has been emphasized in other mentoring research, specifically in Varga and Zaff’s (2018) webs of support framework. Particularly, the webs of support framework outlines the value of important people in a young person’s life working with one another for the betterment of the young person, not just in their own silos. Further, this finding highlights the importance of utilizing the socio-ecological model to understand firearm violence prevention, specifically in regard to all of the systems mentors and CVI programs have to help the youth navigate in order to best serve them and prevent firearm violence (Gillum et al., 2024).

All of the participants identified macro issues that impacted firearm violence for the young people they served. Particularly, participants talked about the impact of racism, the broader culture of violence, toxic masculinity, generational racial trauma, lack of economic resources and opportunities, unaddressed trauma and mental health concerns, social media, and music as factors affecting their young person’s being impacted by firearm violence in some capacity. These macro issues have been brought up in other research studies as well (Bottiani et al., 2021; Buggs et al., 2023; Sanchez et al., 2020; Schleimer et al., 2024).

Two specific topics that participants identified as being unique generational factors for young people and firearm violence were social media and music. Aligning with participants’ insights on the impact of social media on the young people they mentored, research illustrates that social media can be used to amplify conflicts that have the potential to lead to firearm violence among young people (Elsaesser et al., 2021; Patton et al., 2014). Additionally, previous research illustrates the impact of music, specifically hip-hop/rap music, on young people and violence, especially young Black men (Evans, 2020). Specifically, researchers acknowledge the violent nature of some hip hop/rap music, but also highlight that many hip hop/rap artists are simply providing a reflection on their disinvested environments (Evans, 2020). More research on how mentors in CVI programs is needed to explore how mentors handle these topics in the mentoring relationships, especially mentors who may not have a heavy presence on social media or listen to current hip hop/rap music.

## Limitations and Future Research

First, due to the sample size and the demographic of the participants in this study, current findings may not be generalizable to other types of mentoring interventions that utilize a different demographic of mentors or take place in different parts of the United States. Second, the study did not capture the perspectives of young people, particularly Black young people, in these mentoring relationships within CVI programs. Additionally, social desirability bias could have come into play with the responses from the participants in this study. Finally, this study only utilized one coder of the transcripts, and it is essential to consider the ways in which the first author’s positionality impacted the analysis of the data (Jacobson & Mustafa, 2019). Future research can build upon the findings of this study by interviewing both mentors and mentees in CVI programs in different geographic locations across the United States and comparing and contrasting the cultural and generational factors to continue to refine the preliminary framework. Finally, these findings provide key practical implications for CVI programs that can be used to inform the future design and implementation of CVI programs, specifically policies for recruiting, training, retaining, and supports provided to mentors striving to prevent firearm violence.

## Conclusions

Using the perspectives of CVI program mentors and administrators, this study illustrates the components in mentoring interventions within CVI programs they perceived as essential in preventing firearm violence among young people. Specifically, program mentors and administrators stressed that CVI programs must prioritize recruiting talented mentors with shared lived experiences of young people, providing a consistent and stable relationship during the mentoring intervention, addressing the basic needs of young people and their families, coordinating with different people across different systems that impact the young person, teaching young people relevant life lessons and skills to navigate their environment safely, and acknowledging the macro factors that lead to firearm violence. Participants emphasized the incredibly complex and demanding work being implemented in mentoring interventions within CVI programs and stressed not everyone is able to be a mentor in their community, as one participant (#10) stated:

…There’s a lot of folks around the community that look at us and think they can do what we do, and they just think that it’s easy… one thing I try to show people or tell people, when you see a professional doing something, it usually looks easy, because they’re professional and they’re doing it.

Ultimately, the perspectives of mentors and administrators illustrate the components of mentorship interventions within CVI programs that have the potential to reduce firearm violence in their communities, especially among Black young people and their families.

## Data Availability

Interview guides and qualitative summaries are available upon request to the corresponding author with completion of a data sharing agreement.

## References

[CR1] Barrett, J. T., Lee, L. K., Monuteaux, M. C., Farrell, C. A., Hoffmann, J. A., & Fleegler, E. W. (2022). Association of county-level poverty and inequities with firearm-related mortality in US youth. *JAMA Pediatrics,**176*(2), Article e214822. 10.1001/jamapediatrics.2021.4822 34807238 10.1001/jamapediatrics.2021.4822 PMC8609463

[CR2] Bottiani, J. H., Camacho, D. A., Lindstrom Johnson, S., & Bradshaw, C. P. (2021). Annual research review: Youth firearm violence disparities in the United States and implications for prevention. *Journal of Child Psychology and Psychiatry,**62*(5), 563–579. 10.1111/jcpp.13392 33797082 10.1111/jcpp.13392 PMC9993333

[CR3] Buggs S. (2022). *Community-based violence interruption and public safety*. Arnold Ventures. https://craftmediabucket.s3.amazonaws.com/uploads/AVCJIReport_Community-BasedViolenceInterruptionPublicSafety_Buggs_v2.pdf

[CR4] Buggs, S. A. L., Kravitz-Wirtz, N. D., & Lund, J. J. (2023). Social and structural determinants of community firearm violence and community trauma. *The ANNALS of the American Academy of Political and Social Science,**704*(1), 224–241. 10.1177/00027162231173324(Originalworkpublished2022)

[CR5] Butts, J. A., Roman, C. G., Bostwick, L., & Porter, J. R. (2015). Cure violence: A public health model to reduce gun violence. *Annual Review of Public Health,**36*(1), 39–53. 10.1146/annurev-publhealth-031914-122509 25581151 10.1146/annurev-publhealth-031914-122509

[CR6] Center on the Developing Child at Harvard University (2021). Three principles to improve outcomes for children and families, 2021 Update. https://www.developingchild.harvard.edu. Retrieved March 30, 2025.

[CR7] Charmaz, K. (2014).* Constructing grounded theory (2nd ed.). SAGE Publications.*

[CR8] Cheng, T. L., Wright, J. L., Markakis, D., Copeland-Linder, N., & Menvielle, E. (2008). Randomized trial of a case management program for assault-injured youth: Impact on service utilization and risk for reinjury. *Pediatric Emergency Care,**24*(3), 130–136. 10.1097/PEC.0b013e3181666f72 18347488 10.1097/PEC.0b013e3181666f72

[CR9] Corburn, J., Boggan, D., Muttaqi, K., Vaughn, S., Houston, J., Thibodeaux, J., & Muhammad, B. (2021). A healing-centered approach to preventing urban gun violence: The advance peace model. *Humanities and Social Sciences Communications,**8*(1), Article 142. 10.1057/s41599-021-00820-y

[CR10] Elsaesser, C., Patton, D. U., Weinstein, E., Santiago, J., Clarke, A., & Eschmann, R. (2021). Small becomes big, fast: Adolescent perceptions of how social media features escalate online conflict to offline violence. *Children and Youth Services Review,**122*, Article 105898.

[CR11] Evans, J. (2020). We [mostly] carry guns for the internet’: Visibility labour, social hacking and chasing digital clout by Black male youth in Chicago’s drill rap scene. *Global Hip Hop Studies,**1*(2), 227–247.

[CR12] Gillum, T. L., Hampton, C. J., & Coppedge, C. (2024). Using the socio-ecological model to understand increased risk of gun violence in the African American community. *Psychological Reports*. 10.1177/00332941241256635 38804858 10.1177/00332941241256635

[CR13] Goldner, L., & Ben-Eliyahu, A. (2021). Unpacking community-based youth mentoring relationships: An integrative review. *International Journal of Environmental Research and Public Health,**18*(11), 5666. 10.3390/ijerph18115666 34070652 10.3390/ijerph18115666 PMC8198211

[CR14] Hureau, D. M., & Papachristos, A. V. (2024). Re-Centering the community in violence intervention: Reclaiming legacies of street outreach in the provision of public safety. *Annual Review of Criminology*, *8*. 10.1146/annurev-criminol-030920-085949

[CR15] Jackson-Spierker, K., & Barrows, A. (2024). Experts by experience: How engaging people with lived experience can improve social services. Retrieved from https://projectevident.org/wp-content/uploads/2023/12/CBDSJ-Experts-by-Experience-Jan2024.pdf

[CR16] Jacobson, D., & Mustafa, N. (2019). Social identity map: A reflexivity tool for practicing explicit positionality in critical qualitative research. *International Journal of Qualitative Methods*. 10.1177/1609406919870075

[CR17] Jones, V., Becote-Jackson, M., Parnham, T., Lewis, Q., & Ryan, L. M. (2021). Violence prevention through mentoring for youth with emergency department treated peer assault injuries. *The Journal of Pediatrics,**6*, Article 100064. 10.1016/j.ympdx.2021.100064 10.1016/j.ympdx.2021.100064 PMC10236538 37333430

[CR18] Lester, A. M., Goodloe, C. L., Johnson, H. E., & Deutsch, N. L. (2019). Understanding mutuality: Unpacking relational processes in youth mentoring relationships. *Journal of Community Psychology,**47*(1), 147–162. 10.1002/jcop.22106 30506928 10.1002/jcop.22106

[CR19] Mariño-Ramírez, L., Jordan, I. K., Nápoles, A. M., & Pérez-Stable, E. J. (2022). Comparison of US firearm-related deaths among children and adolescents by race and ethnicity, 1999–2020. *JAMA,**328*(23), 2359–2360. 10.1001/jama.2022.19508 36538317 10.1001/jama.2022.19508 PMC9832380

[CR20] Masten, C., Lombardi, J., & Fisher, P. (2021). Helping families meet basic needs enables parents to promote children’s healthy growth, development. *Center on Budget and Policy Priorities.* Retrieved March, 30, 2025.

[CR21] Patton, D. U., Hong, J. S., Ranney, M., Patel, S., Kelley, C., Eschmann, R., & Washington, T. (2014). Social media as a vector for youth violence: A review of the literature. *Computers in Human Behavior,**35*, 548–553. 10.1016/j.chb.2014.02.043

[CR22] Pulavarthi, T. S., Fabio, A., Miller, E., & Culyba, A. J. (2024). Examining associations between school connectedness, social support, violence, and firearm carrying. *Journal of Interpersonal Violence,**39*(15–16), 3651–3668. 10.1177/088626052412332673211235 38379210 10.1177/08862605241233267

[CR23] Sanchez, C., Jaguan, D., Shaikh, S., McKenney, M., & Elkbuli, A. (2020). A systematic review of the causes and prevention strategies in reducing gun violence in the United States. *The American Journal of Emergency Medicine,**38*(10), 2169–2178. 10.1016/j.ajem.2020.06.062 33071102 10.1016/j.ajem.2020.06.062

[CR24] Schleimer, J. P., Lyons, V. H., Smith, D., Ali, F., Averett, L., Baugh, M., Benson, L. R., Colon, J., Cook, J., Davis, D., Diandy, M., Fox, A., Gonzalez, E., Johnson, A., Lowe, A. B., Marshall, M., Maryman, B., McLaurin, V., Nehra, D., … Rowhani-Rahbar, A. (2024). Codeveloping theories of change for improved community-based violence intervention evaluation. *Journal of Trauma and Acute Care Surgery,**97*(2), 278–285. 10.1097/TA.0000000000004277 38509040 10.1097/TA.0000000000004277

[CR25] Spencer, R., Pryce, J., Barry, J., Walsh, J., & Basualdo-Delmonico, A. (2020). Deconstructing empathy: A qualitative examination of mentor perspective-taking and adaptability in youth mentoring relationships. *Children and Youth Services Review,**114*, Article 105043. 10.1016/j.childyouth.2020.105043

[CR26] Swanson, J. W., Tong, G., Easter, M. M., Sivaraman, J. C., Gifford, E. J., Gardner, B. O., Donnelly, E. A., Evans, K. E., Copeland, W. E., Swartz, M. S., & Bonnie, R. J. (2022). Gun violence among young adults with a juvenile crime record in North Carolina: Implications for firearm restrictions based on age and risk. *Preventive Medicine,**165*(Pt A), 107279–107279. 10.1016/j.ypmed.2022.107279 36191654 10.1016/j.ypmed.2022.107279

[CR27] Varga, S. M., & Zaff, J. F. (2018). Webs of support: An integrative framework of relationships, social networks, and social support for positive youth development. *Adolescent Research Review,**3*(1), 1–11. 10.1007/s40894-017-0076-x

[CR28] Wical, W., Richardson, J., & Bullock, C. (2020). A credible messenger: The role of the violence intervention specialist in the lives of young Black male survivors of violence. *Violence and Gender,**7*(2), 66–69. 10.1089/vio.2019.0026

[CR29] Wolf, E. R., Rivara, F. P., Orr, C. J., Sen, A., Chapman, D. A., & Woolf, S. H. (2024). Racial and ethnic disparities in all-cause and cause-specific mortality among US youth. *JAMA,**331*(20), 1732–1740. 10.1001/jama.2024.3908 38703403 10.1001/jama.2024.3908 PMC11070063

[CR30] Zheng, N., Abram, K. M., Welty, L. J., Aaby, D. A., Meyerson, N. S., & Teplin, L. A. (2023). Nonfatal firearm injury and firearm mortality in high-risk youths and young adults 25 years after detention. *JAMA Network Open,**6*(4), e238902–e238902. 10.1001/jamanetworkopen.2023 37083667 10.1001/jamanetworkopen.2023.8902 PMC10122168

